# Friction Stir Welding of Aluminum in the Aerospace Industry: The Current Progress and State-of-the-Art Review

**DOI:** 10.3390/ma16082971

**Published:** 2023-04-08

**Authors:** Mohamed M. Z. Ahmed, Mohamed M. El-Sayed Seleman, Dariusz Fydrych, Gürel Çam

**Affiliations:** 1 Department of Mechanical Engineering, College of Engineering at Al Kharj, Prince Sattam Bin Abdulaziz University, Al Kharj 11942, Saudi Arabia; 2 Department of Metallurgical and Materials Engineering, Faculty of Petroleum and Mining Engineering, Suez University, Suez 43512, Egypt; 3 Institute of Machines and Materials Technology, Faculty of Mechanical Engineering and Ship Technology, Gdańsk University of Technology, Gabriela Narutowicza Street 11/12, 80-233 Gdańsk, Poland; 4 Department of Mechanical Engineering, Iskenderun Technical University, Iskenderun 31200, Hatay, Türkiye

**Keywords:** friction stir welding, aerospace industry, SSFSW, BTFSW, RFSSW, aluminum–lithium alloys

## Abstract

The use of the friction stir welding (FSW) process as a relatively new solid-state welding technology in the aerospace industry has pushed forward several developments in different related aspects of this strategic industry. In terms of the FSW process itself, due to the geometric limitations involved in the conventional FSW process, many variants have been required over time to suit the different types of geometries and structures, which has resulted in the development of numerous variants such as refill friction stir spot welding (RFSSW), stationary shoulder friction stir welding (SSFSW), and bobbin tool friction stir welding (BTFSW). In terms of FSW machines, significant development has occurred in the new design and adaptation of the existing machining equipment through the use of their structures or the new and specially designed FSW heads. In terms of the most used materials in the aerospace industry, there has been development of new high strength-to-weight ratios such as the 3rd generation aluminum–lithium alloys that have become successfully weldable by FSW with fewer welding defects and a significant improvement in the weld quality and geometric accuracy. The purpose of this article is to summarize the state of knowledge regarding the application of the FSW process to join materials used in the aerospace industry and to identify gaps in the state of the art. This work describes the fundamental techniques and tools necessary to make soundly welded joints. Typical applications of FSW processes are surveyed, including friction stir spot welding, RFSSW, SSFSW, BTFSW, and underwater FSW. Conclusions and suggestions for future development are proposed.

## 1. Introduction

The materials used in aerospace applications are numerous, starting from metallic, ceramic, polymeric, and composite materials [1]. The primary metallic materials used in the aerospace industry include but are not limited to aluminum alloys [2], magnesium alloys [3,4,5,6,7,8,9], titanium alloys [10,11,12,13,14], steel alloys, and Ni-based superalloys. These materials can be classified into low-softening-temperature materials (aluminum and magnesium alloys) and high-softening-temperature materials (nickel-based superalloys, steel alloys, and titanium alloys). During the manufacturing processes of aerospace structures, all types of materials require welding and joining at the highest quality possible. Friction stir welding (FSW) has been proven to satisfy the required quality of welding different types of materials, especially those with low-softening-temperature alloys [15,16,17,18,19,20], and is relatively applied in high-softening-temperature materials such as titanium alloys [21,22,23,24,25] and steel alloys [26,27,28,29,30,31,32,33,34,35]. This is mainly because the available tool materials can be used satisfactorily to produce very long-distance joints without any significant degradation [36,37,38]. FSW’s invention was driven by the need to join the high-strength aluminum alloy series 7xxx and 2xxx, known as non-weldable aluminum alloys, using conventional welding techniques [39]. FSW has progressed and is used in many industrial applications such as marine, railway, automotive, and aerospace [2,40,41,42,43,44,45,46,47]. FSW has been progressively adopted in aerospace applications for welding structures made from high-strength aluminum alloys such as large-volume fuel tanks [45]. Fuel tanks for Delta II and Delta IV rockets were the first significant aerospace applications to use the FSW process to replace the fusion welding techniques [2]. Boeing (the manufacturer) has reported high-cost savings over the previous variable polarity plasma arc (VPPA) process and almost zero defect incidence [2]. In the replacement of the existing rivets in many structures, major airframe manufacturers are investigating the use of FSW [48,49]. The Eclipse 500 business jet was one of the first aircraft to adapt FSW technology in its upper and lower wing skins, cabin skins, side cockpit skins, engine beam, and aft fuselage skins [48]. FSW technology enables faster joining with speeds up to sixty times faster than manual riveting or six times faster than automated riveting with improved quality, resulting in a significant cost reduction. The assembly of the Eclipse 500 using FSW required the design, development, and fabrication of a custom, high-performance FSW system with manipulation and process control capabilities beyond what had previously been produced by the FSW system [48]. NASA’s Space Launch System (SLS) used FSW to manufacture a 39 m-long liquid hydrogen tank using a giant 52 m-tall friction stir welding facility specially built for the SLS [50,51,52,53]. The SLS main stage also comprises a liquid oxygen tank, an aft engine section, an intertank section, and a forward skirt. The thickest aluminum structures ever assembled used friction stir welding in the SLS core stage [50,51,52,53]. Recently, Indian Space Research Organization (ISRO) launched a rocket in 2018, the first to fly with propellant tanks constructed using FSW, and claimed that FSW is a more efficient manufacturing method to improve the productivity and payload capability of the vehicle [18]. In terms of high-softening-temperature materials, although the tool materials still limit the wide industrial applications of the FSW process, it has also progressed in some applications, such as in the use of the cast Ti-6Al-4V in the manufacture of the spacecraft propellant tank, aimed at reducing lead time and costs compared to the routes of conventional manufacturing [42]. FSW has numerous advantages over other solid-state methods of severe plastic deformation with the purpose of bonding or joining, such as accumulative roll bonding [54,55,56]. FSW can be used to produce different configuration joints such as butt [57], lap [58], T [37] and corner joints [59]. Recently, FSW principles have been adopted for additive manufacturing in the solid state as well as in different configurations [60].

The fuel tanks of space shuttles and spaceships have been manufactured from welded structures of high-strength aluminum alloys. These welded structures usually experience a complex internal/external pressure and structure torque during the service, which requires high-standard welds [45]. The use of conventional fusion welding has commonly resulted in porosities and hot cracking in the joined structures. On the other hand, FSW has attracted extensive interest from the aerospace industry owing to its exceptional advantages including fewer defects [45]. Boeing has reported virtually zero defect incidence and significant cost savings over the previous variable polarity plasma arc (VPPA) process [2], as well as low distortion and excellent joint performance [45]. Thus, FSW has been accepted as an ideal technique for joining large aerospace structures made of high-strength aluminum alloys [2,45], and has been investigated and optimized for the welding of titanium alloys and stainless steel alloys [61].

**Figure 1:**
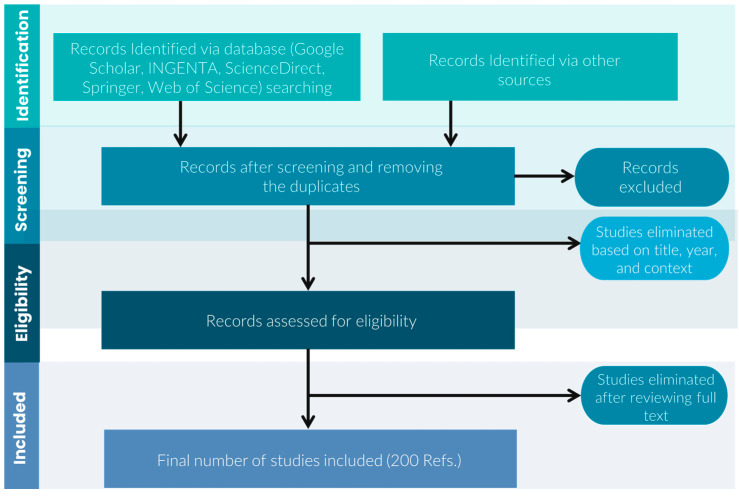
A schematic flowchart of the strategy used to prepare the current review.

**Figure 2:**
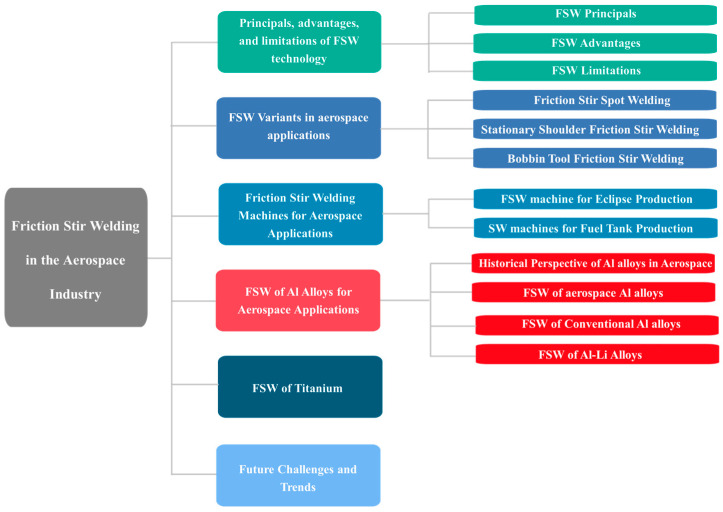
The structure of the current review.

As described above, there has been significant progress on the FSW process, but only limited literature can be found regarding a more comprehensive review of FSW in the aerospace industry. With this background, we tried to provide a review of the historical development of FSW technology followed by the state of the art of FSW for aerospace applications. A literature survey was conducted in the Web of Science and Scopus databases and the Google Scholar internet search engine based on the terms: “FSW + aerospace” and “friction stir welding + aerospace”. Due to the novelty of the FSW subject (not longer than 30 years), the search was not limited by time. This resulted in a collection of over 300 articles. After removing duplicate, substantively distant articles and those of dubious quality (e.g., unpublished and unreviewed research reports), almost 200 papers were left for further analysis. Figure 1 shows a schematic flowchart of the strategy used to prepare the current review. This review reports the principals of FSW, its advantages, and limitations concerning aerospace applications in Section 2. The main FSW variants applied in the aerospace industry, such as friction stir spot welding, stationary shoulder friction stir welding, and bobbin tool friction stir welding, are outlined in Section 3. The FSW production machines in the aerospace industry are described in Section 4, mainly the Eclipse FSW machine and fuel tank FSW machines. The up-to-date research in the FSW of aerospace materials is summarized in Section 4 with a focus on aluminum alloys. Figure 2 shows the structure of this review.

## 2. FSW Principals, Advantages, and Limitations

### 2.1. FSW Principals

Friction stir welding (FSW) is currently considered a well-developed solid-state joining process that was invented by The Welding Institute (TWI) in 1991 [62,63,64], mainly for the purpose of joining the aerospace aluminum alloys 2xxx and 7xxx series of relatively high strength, which at the time were known to be non-weldable due to both porosity formation in the fusion zone and the poor solidification microstructure, and thus poor mechanical properties, as is the case for other high strength aluminum alloys [65,66,67,68,69,70,71,72,73]. Since then, FSW has been rapidly developed into a doable joining technology for a range of metals and alloys, and is used in applications from microelectronics to space shuttles [74].

**Figure 3:**
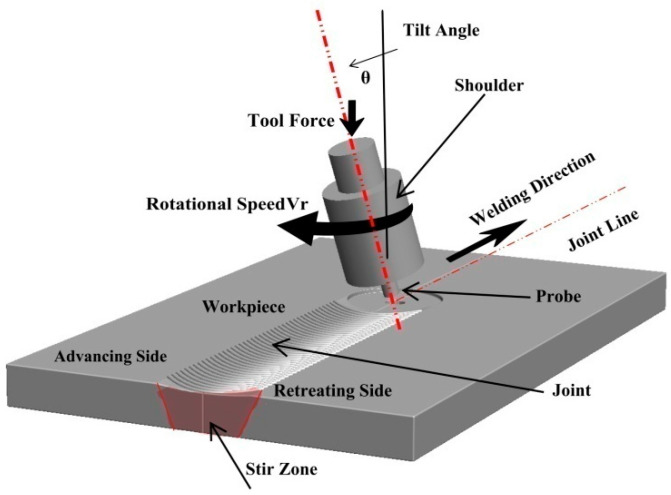
A schematic of the FSW process, indicates the process’s main characteristic features. The FSW tool is shown with the exit hole just below the tool after extraction.

**Figure 4:**
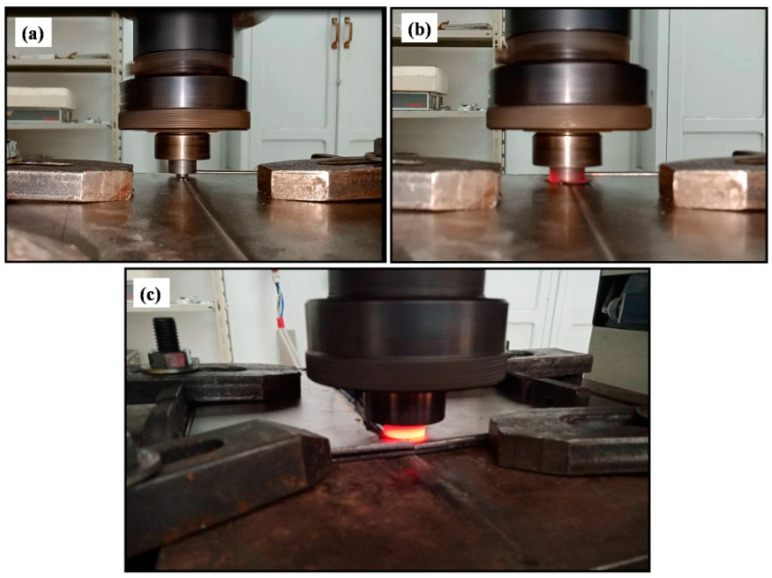
Images showing the stages of FSW steel alloy using the WC tool. (**a**) Initial stage of a tool plunging at the abutting edge of the two tightly clamped plates on the table of the FSW machine, (**b**) after plunging and traversing, and (**c**) at the end of the FSW and just before extracting the tool.

The FSW innovation was in the use of an external non-consumable rotating tool to accomplish the welding in the solid state. The tool as the key player in the FSW process consists mainly of a shoulder and a probe (pin). The ratio between the shoulder (larger diameter) and the probe (smaller diameter) depends mainly on the type and thickness of the welded material [75] and sometimes on the type of tool material [76]. To conduct FSW, this tool, while rotating at a predetermined rotation rate (rpm), is plunged into two abutting or overlapped plates or sheets until achieving full penetration of the probe with enough pressure from the shoulder on the top surface of the plates. This will heat and cause the softening of the materials around the tool in this area and make its plastic deformation possible and steady. At this stage, the tool can traverse with a predetermined speed (mm/min) along the joint line to produce the joint in a solid state. During FSW, the constrained soft material around the tool is moved from the advancing side (in which the tool traversal direction and tool rotation direction are similar) to the retreating side (in which the tool traversal direction and tool rotation direction are opposite). This sequence of actions will build the joint area behind the tool at a rate that depends on the ratio between the tool rotation rate and traversal speed. At the end of the predetermined joint length, the tool exists while rotating, leaving the keyhole behind, which is known to be one of the characteristic features of FSW. Figure 3 shows a schematic of the FSW process in which all the FSW-related terms are indicated. This schematic shows the FSW tool after exit, and the tilt angle is exaggerated for clarity. Figure 4 shows images of the FSW stages of a steel alloy using a WC tool. The tool, while rotating, can be seen plunging between the abutting plates in (a), and then after the complete plunging of the tool and just before traversing along the joint line in (b), and at the end of the welding pass and just before extraction in (c).

**Figure 5:**
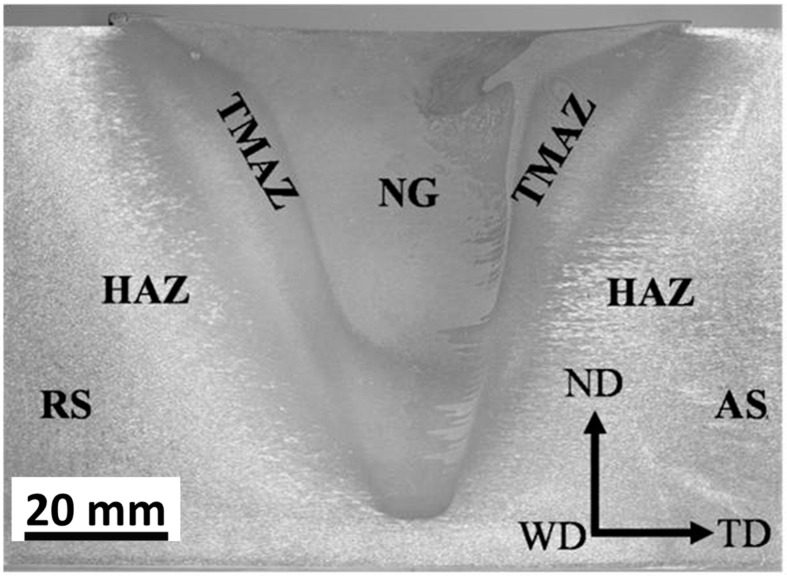
Transverse cross-section optical macrograph of friction-stir-welded 75 mm-thick AA6082 on which the different zones are labeled. TD, WD, and ND stand for transverse direction, welding direction, and normal direction, respectively.

The weld area of the friction-stir-welded materials has some characteristic features that distinguish the FSW weld area from the weld areas of other joining techniques. It consists of four main zones: (1) The base material (BM) represents the part of the material that neither experiences any plastic deformation nor enough heat, so all the microstructural features and properties of the BM are preserved. (2) The heat-affected zone (HAZ) represents the second zone towards the center of the weld area that does not experience any plastic deformation, and only enough heat to affect some microstructural features and their dependent properties. The width of the HAZ and the effect of the thermal cycle on its microstructural constituents mainly depend on the heat input experienced during the welding process and the type of welded material. (3) The thermo-mechanically affected zone (TMAZ) represents the third zone towards the center of the weld area that experiences both plastic deformation and enough heat to distort the grain structure due to the passage and the shear effect of the tool. The TMAZ is highly affected in terms of heat but slightly in terms of deformation, which is why it represents the weakest area in terms of the hardness of the FSWed heat-treatable aluminum alloys, and failure always occurs at the TMAZ. (4) The stir zone or the nugget zone (NG) represents the central zone of the weld area where the highest heat and plastic deformation occurs due to the continuous stirring of the softened material around the FSW tool. The NG, due to this severe thermo-mechanical process, undergoes significant microstructural changes represented by the formation of a completely new microstructure, either due to the recrystallization processes and/or phase transformation processes that take place at the high strain rate, temperature, and strain based on the type of welded material. Figure 5 shows the transverse cross-section optical macrograph of friction-stir-welded 75 mm-thick AA6082, on which the HAZ, TMAZ, and NG zones can clearly be observed. It can be noted that the interface between the NG and the TMAZ at the AS is quite sharp, while at the RS it is diffusive.

**Figure 6:**
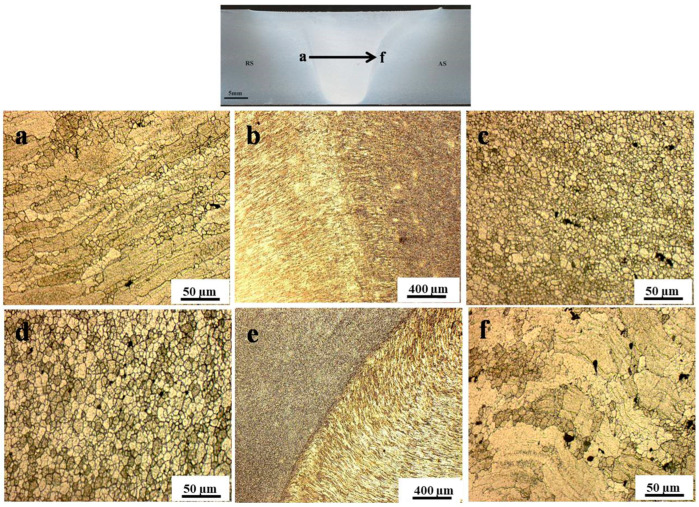
Optical microstructure across the weld area along the indicated arrow in the optical macrograph of FSWed AA7075-T6. (**a**,**b**) TMAZ–NG RS interface, (**c**,**d**) NG, and (**e**,**f**) TMAZ–NG AS interface.

The optical microstructure across the different zones of the weld area in FSWed 20 mm-thick AA7075 is presented in Figure 6a–f. The microstructure sequence is shown according to the arrow indicated on the optical macrograph of the joint above the figure. Figure 6a,b clearly shows the diffusive interface and the rotated large grain structure in the TMAZ at the interface between the TMAZ and the NG at the RS. Figure 6c,d, inside the NG zone, indicates the recrystallized grain structure, and it can be observed that the grain size is slightly more prominent at the AS (d) than at the RS (c) due to the height of the AS. Figure 6e,f clearly shows the sharp transition and the rotated large grain structure at the TMAZ at the interface between the TMAZ and the NG at the AS. These macro- and microstructural features of the FSWed materials have a strong implication for the enhancement of properties and the integrity of joints.

### 2.2. Advantages of FSW

FSW is a solid-state process that has many advantages to be used in aerospace applications [2,65]:

1. The weld nugget experiences a high-strain-rate plastic deformation process at a relatively high temperature, resulting in a dynamically recrystallized structure that, in most of the alloys, is a refined grain structure.
2. The weld zone experiences low heat input, resulting in low distortion in the welded plates.
3. It is a fully automated, repeatable process with a limited number of variables involved.
4. Different aerospace materials both in similar and dissimilar configurations can be welded in all kinds of joint configurations.
5. Joints with improved mechanical properties comparable to conventional fusion welding techniques can be fabricated.
6. Significant cost and time savings as the tool is almost non-consumable and the thick sections can be welded in one pass.

### 2.3. Limitations of FSW

There are some limitations to the usage of the FSW process in aerospace applications to be summarized below [2,65]:

1. The workpiece to be welded has to be clamped and strained on top of the backing plate to avoid separation and flowing down the material upon tool plunging and traversing.
2. The machines are not flexible in terms of accessibility, and some parts require manual welding. In addition, the FSW machines are specially designed for specific applications that can cause the capital investment to be high.
3. The tool life for the FSW of high-melting-point materials is still one of the challenges that limit the use of FSW in some applications.

## 3. FSW Variants in Aerospace Applications

### 3.1. Friction Stir Spot Welding

**Figure 7:**
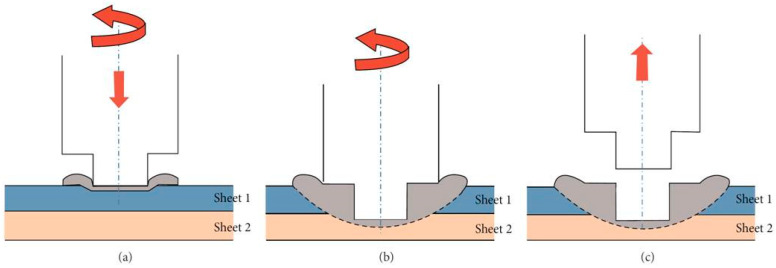
A FSSW process schematic of the tool actions steps. (**a**) Plunging while rotating, (**b**) dwelling after plunging to the specified depth, and (**c**) FSSW tool extraction [85].

**Figure 8:**
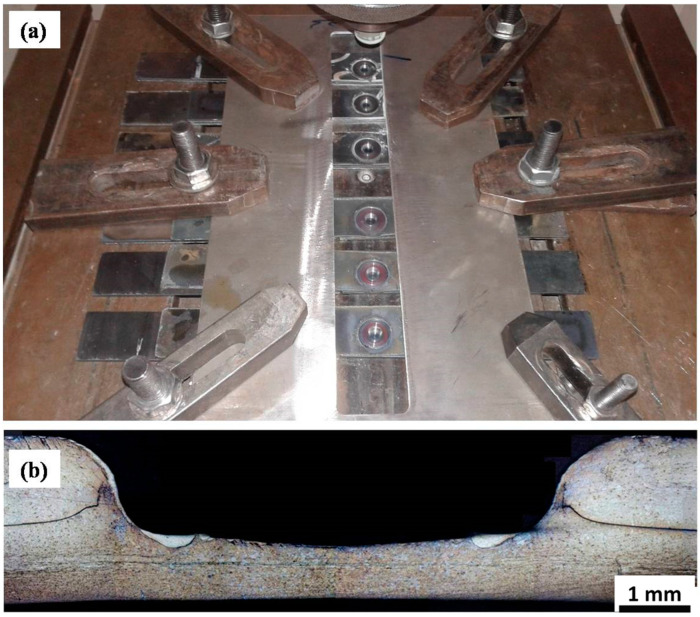
(**a**) Top view of FSSWed TWIP steel sheets showing the exit holes and (**b**) macrograph of the transverse cross-section showing the reduction in the sheet thickness.

**Figure 9:**
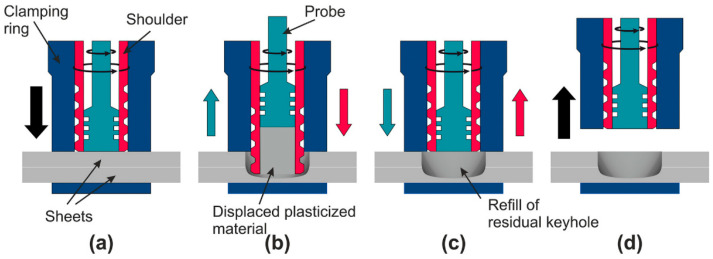
Schematic representation of the shoulder-plunge refill FSSW mode: (**a**) clamping of the sheets, (**b**) shoulder plunging and probe retraction, (**c**) shoulder and probe reaching back to the sheet’s surface and refilling the keyhole, and (**d**) releasing of the clamping force and tool set lifting [80].

Friction stir spot welding (FSSW) is one of the FSW variants developed for local welding applications, mainly to replace riveting in some aerospace applications. The basic principle of FSSW is the same as that of FSW; instead, there is no traversing in this case. In this regard, FSSW consists of three stages: (1) FSSW tool plunging while rotating up to a specified plunge depth, (2) dwelling for a specific time after penetration for the specified plunge depth, and (3) retracting the tool, leaving behind the joint with a keyhole as a characteristic feature [77,78]. Figure 7 shows a schematic of the FSSW process stages, and Figure 8 shows a top view of a series of FSSW points with the exit holes apparent in (a) and the transverse section of the joints presented in (b), which shows the reduction in the thickness in the sheets after FSSW. This process is termed conventional FSSW with some limitations such as the keyhole, thickness reduction of the top sheet, and the presence of “hook” bonding features [79]. To overcome these limitations, a refill FSSW (RFSSW) was developed for aerospace applications by Kawasaki Heavy Industries (KHI) as a new derivative of the conventional FSSW process. RFSSW does not leave an exit hole behind in the workpiece after producing the solid-state lap joint between sheet metals [47]. Figure 9 shows a schematic diagram of the stages of the refill FSSW process [47,80]. For conducting RFSSW, a preheating stage is started while the probe and shoulder are aligned at the same level at the top sheet surface. The stirring friction effect in this stage softens the workpiece, allowing the rotating tool to start plunging in alternating movements between the probe and the shoulder. In the second stage, the shoulder begins to plunge, thus causing more softening of the material, so the plasticized material is injected up into the pin slot. During the third stage, the probe starts plunging to re-inject the displaced material. In the final stage, the shoulder and the probe are aligned parallel to each other again on the top surface to induce a spot joint without a keyhole [47,81,82,83,84].

**Figure 10:**
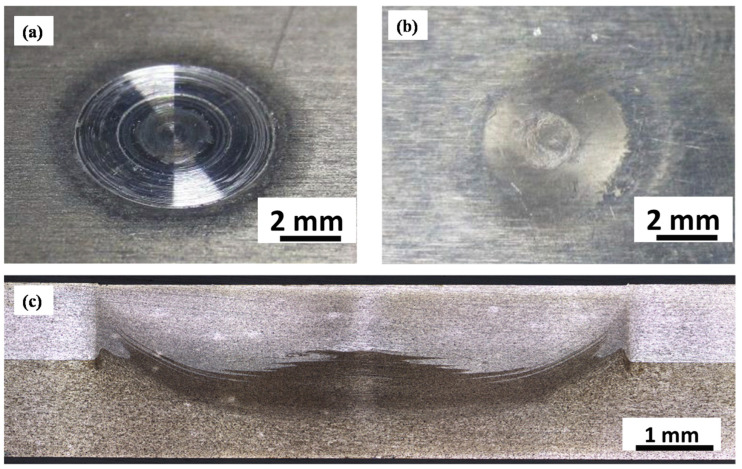
RFSSW joint between AA2024-T3 and AA7075-T6 sheets for aerospace applications. (**a**) Top view, (**b**) bottom view and (**c**) transverse cross-section macrograph [47] (has permission from Elsevier).

**Figure 11:**
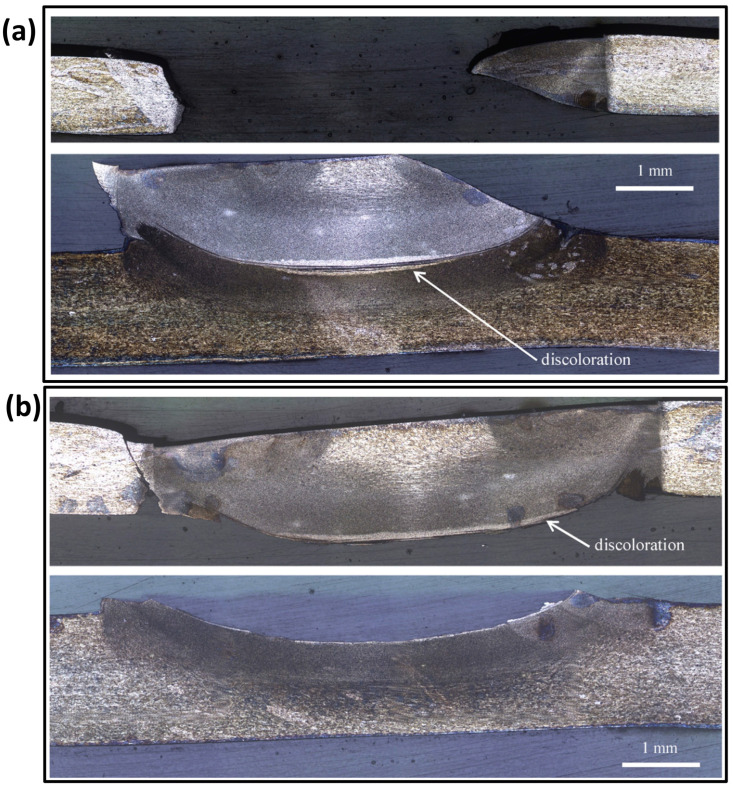
Failure mode of the RFSSWed AA2024-T3 and AA7075-T6. (**a**) Cross-Section of nugget pullout failure: top sheet cross-section (**top**) and bottom sheet cross-section (**bottom**). (**b**) Cross-Section of interfacial failure: top sheet cross-section (**top**) and bottom sheet cross-section (**bottom**). The cross-section plane is parallel to the pull direction [47] (has permission from Elsevier).

The RFSSW technique was used successfully by Boldsaikhan et al. [47] for the dissimilar welding of aerospace aluminum alloys AA7075-T6 and AA2024-T3. In this work, AA2024-T3 was used as the lower sheet representing the skin side of the aircraft structure, and AA7075-T6 was used as the top sheet representing the stiffener side of a skin-stiffener structure of the aircraft. Figure 10 shows the top and bottom view after RFSSW application in (a) and (b), respectively. Figure 10c shows the joint transverse cross-section macrograph where the keyhole is wholly eliminated. In their investigation of the fracture mode of the RFSSWed joints, Boldsaikhan et al. [47] reported two failure modes: a nugget pullout failure as shown in Figure 11a and an interfacial failure as shown in Figure 11b. In terms of failure they reported a load of 5.45 kN for the optimized RFSSW parameters that produced the nugget pullout failure. They also reported that this failure load was substantially greater than the shear load of 2.65 kN of a standard rivet with similar size [47]. This implies that the refilling technique enhances the spot joint quality and strength. Recently, Ahmed et al. [86] developed a refill technique based on friction stir deposition [87,88,89,90,91,92]. They reported that the RFSSW lap joints of AA6082 that were filled with AA2011 showed higher tensile shear loads than those of the FSSW (before refill) lap joints. The RFSSW joint (welded at 600 rpm/3 s and refilled at 400 rpm/1 mm/min) showed a higher tensile shear load of 5400 N ± 100 compared with that recorded by the unfilled joint (4300 N ± 80) [86]. Zu et al. [93,94] investigated the RFSSW of 2.0 mm-thick 2219-O (upper plate) and 2219-C10S (lower plate) with a different thickness. They reported that the lap shear load of the joints fabricated using a lower plate thickness of 4, 10, and 14 mm was 7.4 ± 0.3 kN, 6.7 ± 0.2 kN, and 6.4 ± 0.4 kN, respectively; all of them failed as a plug fracture mode. De Castro et al. [80] investigated the effect of AA2198-T8 RFSSW on tool wear. They performed a total of 2350 welds of AA2198-T8 sheets, and the effect of wear on the probe and shoulder was investigated. While the probe did not suffer any considerable wear after this number of welds, the shoulder underwent wear in different areas, with distinct wear mechanisms. Adhesive wear and plastic deformation were determined as the primary damage mechanisms affecting other shoulder areas. They suggested that the worn shoulder surface reduced the lap shear strength of the joints while all the tested welds surpassed the minimum standard lap shear strength requirements for aeronautical applications [80]. Numerous studies are available in the literature investigating the RFSSW of various aluminum alloys such as AA7075 [95,96,97], AA7050 [98], AA2024 [99,100], AA2198 [80], AA2014 [101], AA6061 [102,103], AA2219 [93], dissimilar Al alloys [102,104], dissimilar Al/steel [105], Mg alloys [9,106,107], dissimilar Mg, and steel [7].

### 3.2. Stationary Shoulder Friction Stir Welding

**Figure 12:**
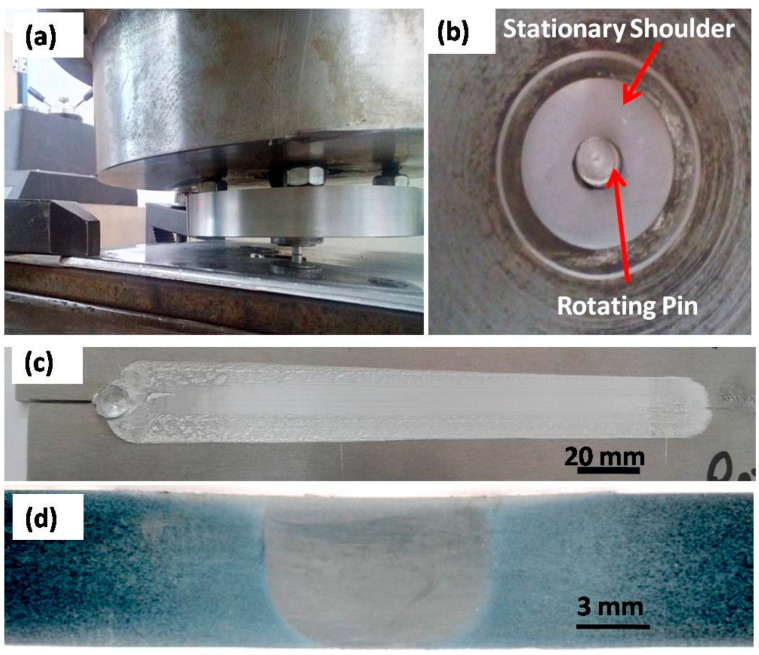
(**a**) External view of SSFSW setup upon plunging. (**b**) Underneath view showing the stationary shoulder and the rotating pin. (**c**) Top view of the SSFSWed AA7075. (**d**) Transverse cross-section macrograph of SSFSWed AA7075 [112].

The FSW of titanium-based alloys such as Ti-6Al-4V, which are used for aerospace applications, has been limited due to their poor thermal conductivity. The heat is mainly generated at the upper surface when conventional FSW tools are used, resulting in a substantial through-thickness temperature gradient. Combined with the limited but relatively high hot working range of alloys such as Ti-6Al-4V, the conventional FSW of titanium is virtually impossible [108]. The TWI has developed the stationary shoulder friction stir welding (SSFSW) variant to overcome this problem and weld the titanium alloys using FSW [109]. In the SSFSW process, the tool pin only rotates through a non-rotating shoulder that only slides over the joint area. Having the shoulder stationary significantly reduces the shoulder contribution to heat generation and affects its distribution through the joint thickness. SSFSW generates highly focused heat input around the tool pin (probe) and eliminates excessive surface heating [110]. Figure 12 shows (a) an external view of the SSFSW setup upon plunging, (b) an underneath view showing the stationary shoulder and the rotating pin, (c) a top view of the SSFSWed AA7075, and (d) a transverse cross-section macrograph of the SSFSWed AA7075. The SSFSW approach was used by Russell et al. [110] in the welding of 6.35 mm-thick Ti-6Al-4V, they reported that the stationary shoulder allows more uniform heating through the thickness; thus, the microstructure is uniform along the whole cross-section. The SSFSW approach has also been used in welding aluminum to develop a through-thickness uniform microstructure and crystallographic texture [111].

**Figure 13:**
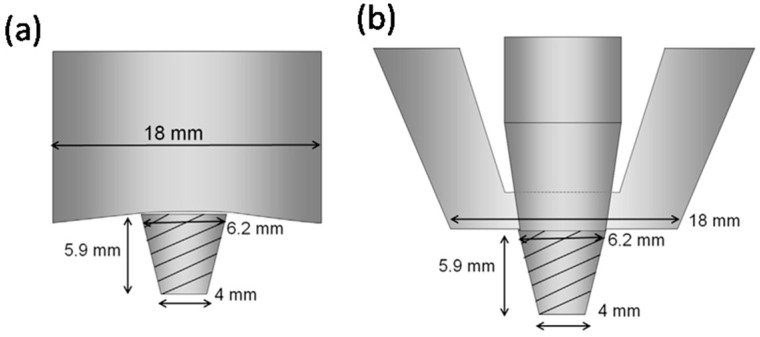
Schematic drawings of the different FSW tools: (**a**) the conventional FSW tool and (**b**) the SSFSW tools used by Wu et al. in their investigation [118] (reprint with permission from Elsevier).

**Figure 14:**
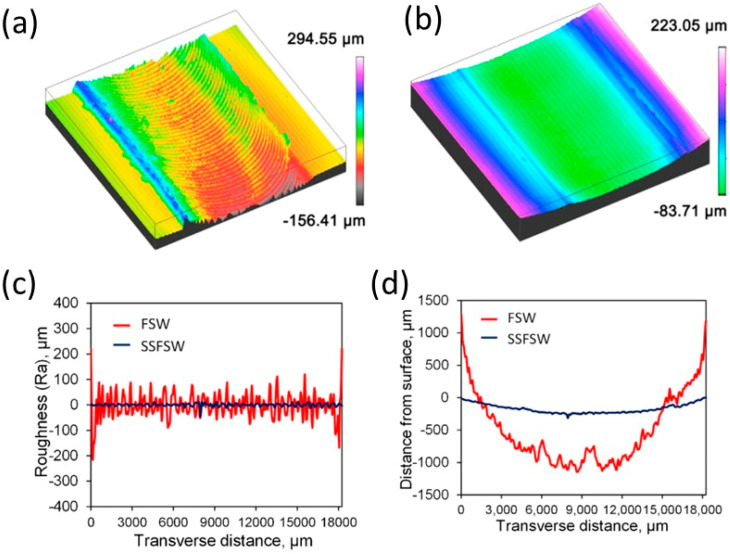
The surface quality characteristics and the surface roughness, respectively, obtained using (**a**,**c**) conventional FSW tool and (**b**,**d**) SSFSW tool [118] (has permission from Elsevier).

The SSFSW approach has been extensively used to join several dissimilar and similar aluminum alloys of high strength [113,114,115,116,117]. Wu et al. [118] carried out a detailed investigation to compare the SSFSW and the conventional FSW of high-strength aerospace AA7050-T765. They used the same pin tool geometries schematically shown in Figure 13 in both FSW and SSFSW, aiming to study the FSW parameters’ effect on the power consumption in each case. They concluded that the welding using SSFSW requires a lower heat input (30%) than that required for the conventional FSW. In addition, the use of the SSFSW resulted in welds with a number of characteristics: (1) narrower heat-affected zone width and a parallel shape; (2) lower through-thickness variation in terms of microstructure and other properties; (3) better cross-sectional tensile properties than the conventional FSW; (4) improvement in the surface roughness due to the non-rotating tool causing ironing on the top surface, as can be noted in Figure 14. On the other hand, they reported that when using too high a tool rotation traversal speed or too high a rotation rate, an adverse effect might occur, such as “speed cracking” that is observed in hot extrusion [118]. These results suggest SSFSW to be one of the promising FSW variants for aerospace applications.

Airbus Group has adopted the SSFSW approach as a significant breakthrough for using the FSW technology in aerospace applications for welding low-melting-point alloys and dissimilar metals, which offers the opportunity for high-quality welds and improvements in production. Airbus Group Innovations, the research and technology arm of Airbus Group, has innovated the DeltaN FS friction stir welding technology, and a stationary shoulder FSW tool system. Mazak machining centers have implemented this tool system to be combined into a range of production processes [119]. The FSW machine’s capital investment is always a significant challenge to using the method in many sectors. Mazak has recently developed and incorporated a combined machine tool and friction stir welding solution into a Vertical Centre Smart 430, a machining center [119] to overcome the high cost of the FSW capital investment. Additionally, Mazak has incorporated the DeltaN FS technology into their range of products, enabling welding functions and machining to be undertaken in the same platform. In the same trend, a new French company has developed an FSW head to be used with the existing machine centers or CN machine tools, drastically reducing the technology cost [119].

**Figure 15:**
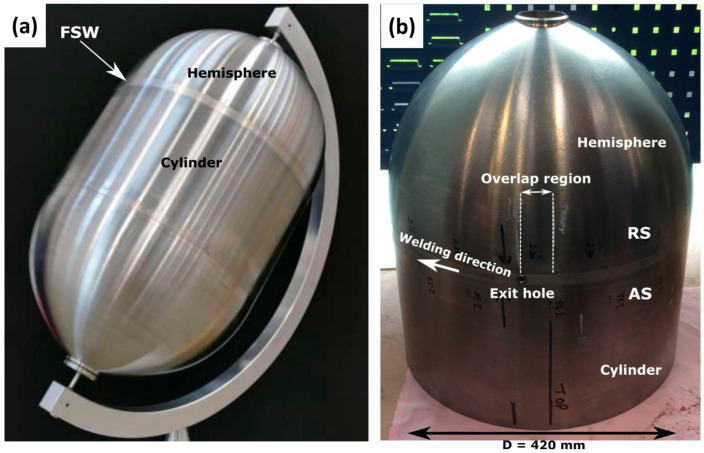
Images of (**a**) prototype of the propellant tank, (**b**) friction stir welded hemicylinder showing overlap region [42] (has permission from Elsevier).

Marie et al. [120] proposed and investigated the use of DeltaN FS technology in joining dissimilar metals for satellite feedthrough. The main function of the feedthrough as a small part of a satellite box is energy supply and transport. Their main specification is vacuum tightness. Feedthroughs are made of dissimilar materials (aluminum alloy/steel or aluminum alloy/titanium) to save weight and facilitate their integration into an aluminum box. To prevent sealant from degassing in space, Marie et al. [120] investigated the use of the stationary shoulder FSW (DeltaN FS) as an alternative to mechanical fastening to join the outer aluminum part around the hard metal connector (steel or titanium) [120]. Additionally, in collaboration with Airbus Defence and Space for the manufacturing of Titanium, TWI has investigated propellant tanks using SSFSW technology for low-cost space applications, and the work was supported by The European Space Agency (ESA) [42]. These tanks used to be manufactured using forging with a combination of fusion welding techniques such as electron beam welding and tungsten inert gas welding (TIG), which require extensive machining that can reach up to a 90% reduction in mass. This makes the propellant tanks one of the most time-consuming and costly items to manufacture for the spacecraft motor system. Thus, the use of FSW in manufacturing these tanks will result in a significant reduction in the time and cost of production in addition to the high-quality joints, which will make FSW a smart option for the future manufacturing of the spacecraft tank. Figure 15a,b shows the propellant tank prototype and the hemicylinder manufactured using FSW.

### 3.3. Bobbin Tool Friction Stir Welding

The use of double-shoulder (upper and lower shoulders) FSW tools, known as bobbin tools, represents one of the new developments of FSW technology that has many advantages in terms of joint quality and machine capabilities, which can be summarized as follows [121,122,123]:

- The full penetration joint eliminates weld root flaws and leads to a lack of penetration defects.
- Low Z forces on fixture and machine.
- Due to the use of the lower shoulder, no backing plate is required.
- Low distortion due to low Z force applied.
- The ability for thickness variation tolerance.
- Capable of joining closed profiles such as hollow extrusions.
- More uniform mechanical properties through the thickness.

**Figure 16:**
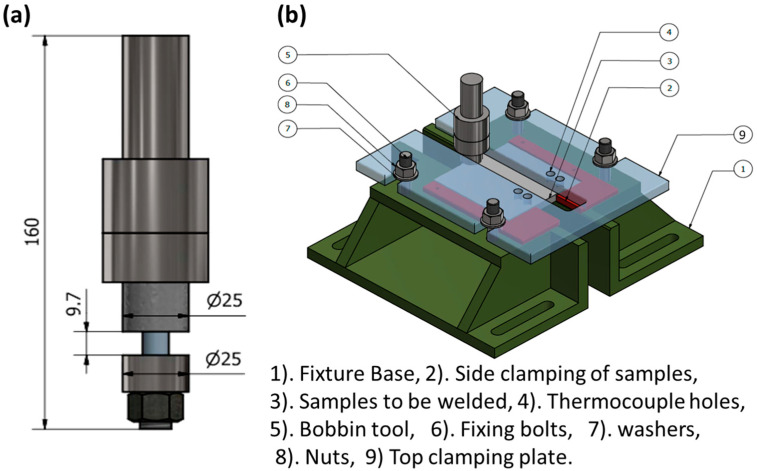
Schematic for (**a**) the FSW bobbin tool for welding 10 mm-thick aluminum, and (**b**) an example of the fixture setup for BTFSW.

Figure 16 shows a schematic for the bobbin tool FSW (BTFSW) tool with the two shoulders (upper and lower) in (a), as well as an example of the fixture setup used for BTFSW presented in (b). It can be mentioned that BTFSW does not need a backing plate, which makes it ideal for welding hollow sections and reduces the applied vertical force that can result in reduced torsion.

The performance of the high-strength aluminum alloys was investigated after welding using BTFSW and compared with that welded by the conventional FSW (CFSW) [121,122,123,124,125,126]. Threadgill et al. [121] investigated the welding of thick sections of AA6082-T6 using both a bobbin tool and a conventional tool and reported that both tools produced sound joints with the difference that the net axial force on the workpiece was almost zero in the case of BTFSW, which has significant beneficial implications in machine design and cost. Xu et al. [124,125] investigated the aluminum alloy AA7085-T7452 after welding using both BTFSW and CFSW. They obtained sound joints in 12 mm-thick sections with lower joint efficiency after BTFSW due to the presence of a Lazy S defect produced by a larger extent of heat input during BTFSW [125]. Yang et al. [126] conducted a comparative study on the use of BTFSW and CFSW in the welding of AA6061-T4. They reported that the strength of the joints produced with BTFSW reached the same level, i.e., about 93%, as that of the CFSW [126]. Wang et al. [122] investigated the BTFSW of aerospace high-strength aluminum alloy AA2198, and they successfully produced sound joints of 3.2 mm thickness using different FSW parameters. The maximum joint efficiency obtained was 80% [122]. Ahmed et al. [58,127,128,129,130] conducted several studies investigating the effect of the tool pin profile and traversal speed mechanical properties of aluminum alloys. Their finding confirmed that the mechanical properties of the base material can be preserved in the weld zone through the optimization of the BTFSW parameters.

The high forces generated during the conventional FSW process that requires backing support underneath the parts to be joined make the FSW system too costly for the aerospace industry due to the very large and varying geometries that require unique large fixtures and support for each. The bobbin tool FSW technology can be the best solution to overcome this limitation. Fraunhofer IWS engineers have adopted the bobbin tool FSW technology to develop an FSW system using flexible fixtures that do not require fixed counter points [44], mainly for welding fuselage structures. This system design does not require additional support structures underneath the joining point, substantially reducing the forces that the machine and the parts must handle. The system developed is a welding robot that autonomously moves on a three-dimensional rail using an internal drive with intelligent clamping that can be suitable for clamping the curved parts. They built a demonstration section size of up to 2.5 m, which was successfully carried out and passed testing [44].

## 4. Friction Stir Welding Machines for Aerospace Applications

### 4.1. FSW Machine for Eclipse Production

First flight of the Eclipse 500 was on 26 August 2002 in which FSW was used to weld the cabin skins, aft fuselage skins, upper and lower wing skins, side cockpit skins and the engine beam [100]. This required the Eclipse Aviation Corporation in collaboration with MTS System Corporation to develop a special FSW system for thin-gauge aerospace aluminum structures with complex contours. It was a gantry type machine with a seven-axis system that was completely instrumented to control and monitor the FSW process. The machine was incorporated with additional sensing systems to ensure the quality of joints. The gantry was selected by MTS from the ones that were available in order to save time in designing and developing a new type. The one that was selected based on their investigation was the U5 gantry from Cincinnati Machine with sufficient stiffness to react the FSW process loads. For this gantry, a welding head was specially designed and built by MTS to allow for the accurate control of the FSW process of complex-contour, thin-gauge applications. The welding head included two rotational axes in addition to the X-, Y- and Z-axes of the gantry motion. One of the welding head axes provided rotation and the other provided pitch. The weld head consisted of a hydraulic spindle motor, a three-degrees-of-freedom load cell, an actuated sensing ring and a patent pending independent spindle axis [100]. To control the pin penetration to ~0.025 mm, a spindle actuator was incorporated to meet one of the main requirements for Eclipse structure manufacturing. The seventh controllable axis of the Eclipse FSW system was the sensing ring. This sensing ring encircled the spindle and was used as a surface for mounting the process monitoring sensors. The controller incorporated within the system was able to monitor the position and load feedback from the redundant sensors and was programmed to abort the process if the signals did not match within an acceptable error band. The communication within Eclipse FSW system does allow the operator to communicate through either a user interface personal computer (PC) or through a remote pendant [100]. 

**Figure 17:**
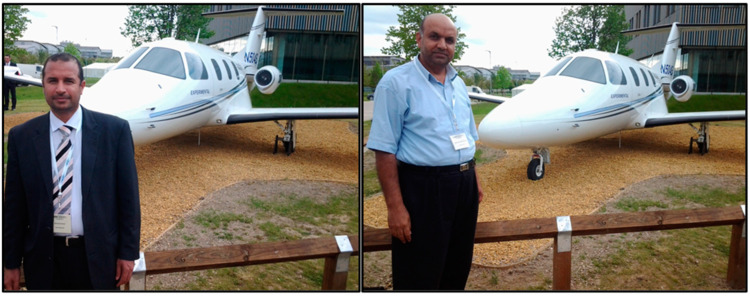
Images for MMZA left and MMES right with the experimental Eclipse 500 business jet presented at TWI–Cambridge as the first aircraft to be manufactured using FSW technology.

Figure 17 shows two images for MMZA left and MMES right with the world’s first production aircraft to use friction stir welding. It was one of the four prototypes used by Eclipse Aerospace during the development and Federal Aviation Administration (FAA) approval of the Eclipse 500. The plane was donated to TWI by Eclipse Aerospace and professionally restored for display by Marshall Aviation Services. The plane was photographed during the 11th International Symposium on Friction Stir Welding, which took place 17–19 May 2016 at TWI in Cambridge.

### 4.2. FSW Machines for Fuel Tank Production

**Figure 18:**
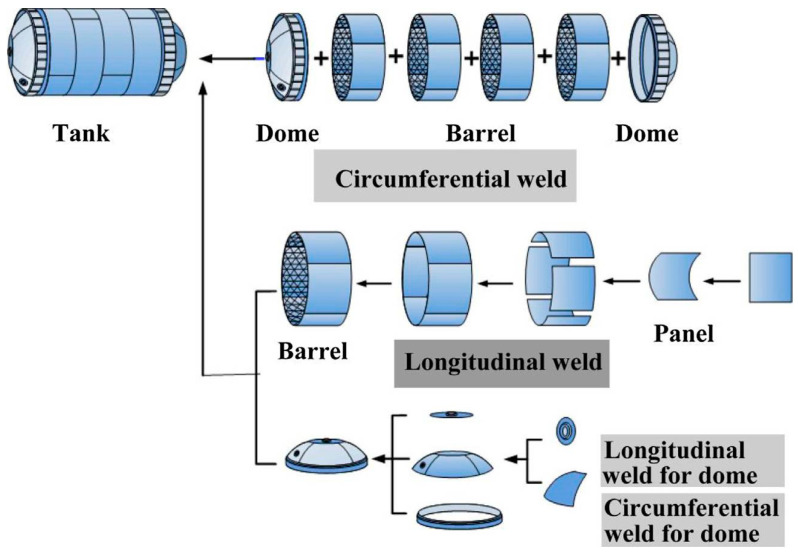
Schematic diagram for structure and main welds of launch vehicle tank [45] (has permission from Elsevier).

For a launching vehicle, spaceship and space shuttle, the fuel tank represents an important structure that mainly consists of a number of cylindrical parts in the middle, top and bottom domes, and one short section at each end as shown schematically in Figure 18 [45]. The requirements for the friction stir welding of fuel tanks are: (1) FSW joints of 3~4 pieces of arc plate sections to form the longitudinal barrel; (2) a variable-curvature longitudinal joint of gores to form the gird; and (3) closed circumferential FSW joints from the gird to the cap, and from the dome to the barrel section, and from section to section [45]. The joint types that are required for the manufacturing of the complete fuel tank include butt joints, variable-curvature joints, closed circumferential joints and lock joints. To manufacture these different types of joints, the FSW machines have to be able to work in two control modes: constant-distance mode and constant-force mode. The constant-force mode is used in the case of the variable-curvature joints. In the constant-force mode, the plunge distance is controlled by keeping the force constant along the joint. This will require a constant-force control unit to be integrated with the welding spindle for the real-time detection of the vertical force. Accordingly, the real-time force is compared with the preset value, which results in the lifting and dropping of the spindle to keep the force constant along the joint profile. The constant-force control mode has been used in FSW for welding the variable-curvature dome [45]. The constant-distance control mode means keeping the plunge distance constant along the joint line, and this is suitable for the straight butt joints such as in welding of the sections and from section to section.

**Figure 19:**
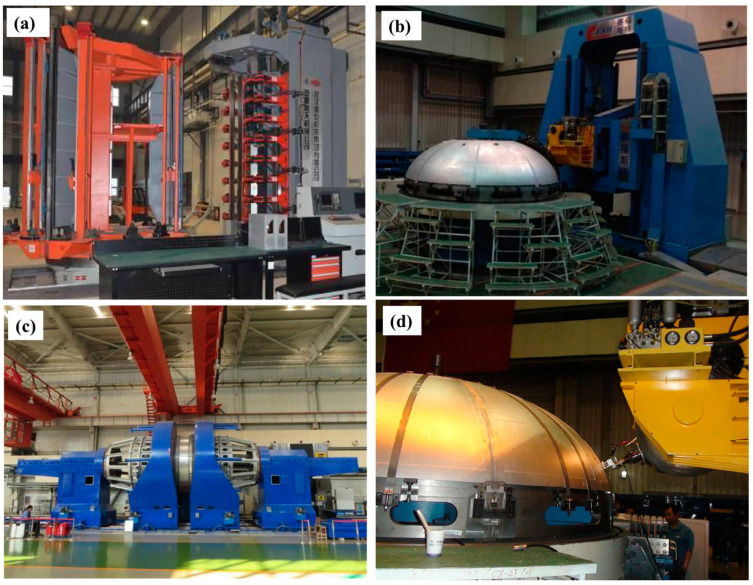
Fuel tank FSW machines. (**a**) Longitudinal FSW equipment of tank section, (**b**) FSW equipment of tank dome, (**c**) circumferential FSW equipment for tank, and (**d**) automatic online PAUT for tank welding with non-planar path [45] (has permission from Elsevier).

Based on that, the FSW machines used in the manufacturing of fuel tanks are classified into three categories: (1) equipment for tank barrel welding, which is a vertical-frame-type longitudinal FSW machine (Figure 19a); (2) equipment for ellipsoid dome welding, which is a large enclosed-frame-type FSW (Figure 19b); (3) circumferential FSW equipment with the integration of outside positioning and main driving (Figure 19c).

**Figure 20:**
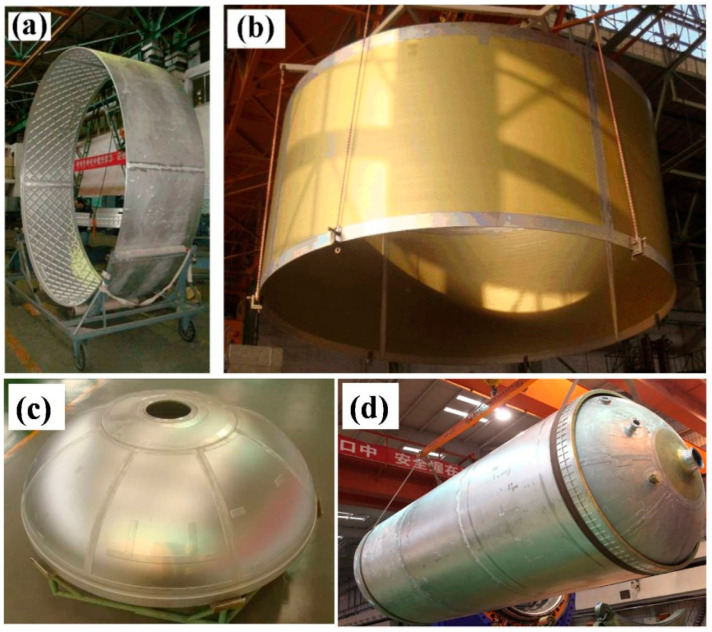
Application of FSW in longitudinal weld of tank section: (**a**) Φ 3350 section; (**b**) Φ 5000 section. (**c**) Application of FSW on Φ 3350 dome. (**d**) Application of FSW on Φ 3350 tank [45] (has permission from Elsevier).

The use of FSW in aerospace applications has resulted in high weld quality and geometry accuracy. In addition, the number of defects that need to be repaired has been reduced from around ~45 to below 3, with a significant high first pass rate. In comparison to fusion welding techniques, the tensile strength of FSW joints has been improved by over 15% from 270~300 MPa to 320~350 MPa. Figure 20 shows some manufactured fuel tank sections of different sizes and geometries.

## 5. FSW of Al Alloys for Aerospace Applications

### 5.1. Historical Perspective of Al Alloys in Aerospace

Al and its alloys possess unique combinations of properties. Thus, Al can be considered one of the most attractive metallic materials for a wide range of applications, from soft, highly ductile foil to highly demanding materials in severe conditions. Al alloys have a long history in aircraft applications. Al was used in aviation before inventing airplanes. In the early nineteenth century, Ferdinand Zeppelin (8 July 1838–8 March 1917) manufactured the frames for his Zeppelin airships from aluminum sheet Al (LZ1–LZ5). On 2 July 1900, Zeppelin made the first flight with the Al-frame LZ1 airship over Lake Constance near Friedrichshafen, southern Germany. The LZ1 airship was developed until reaching the LZ5 airship version, in order to overcome all the accidents that occurred from 1900 to 1910, while retaining the presence of the Al frame as a main component [131,132]. Since then, Al alloys have been of interest in the aerospace industry. During the early nineteenth century (17 December 1903), Al was also chosen as a lightweight material by the Wright brothers for their airplane’s cylinder block and engine components during their first attempt at human flight. This event was accompanied by the first attempt to thermally treat an Al alloy [131,133]. As a result of this discovery, aluminum alloys are preferred in the aerospace industry. A German aircraft designer Hugo Junkers developed the world’s first full-metal aircraft (the Junkers J 1 monoplane) in 1915. Its fuselage was constructed entirely of an aluminum alloyed with magnesium, manganese, and copper [133,134].

Al alloys are widely acceptable for aerospace applications as they possess a light weight, relatively high strength, workability, and corrosion resistance. Besides these advantages, Al base alloys have high availability. Compared to steel, Al is approximately one-third the weight of steel, which enables aircraft to be more fuel efficient and carry greater weight. Steel is utilized in aircraft only when great strength is required, such as in extremely high-speed planes [131,134,135]. The wing panes, the fuselage, the rudder, the exhaust pipes, the floor and door, the seats, the cockpit instruments, and the engine turbines of today’s planes are all made of aluminum. Additionally, all current spacecraft are composed of a 50–90% aluminum alloy. Al alloys were widely employed in the Apollo spacecraft, space shuttles, Skylab space station, and International Space Station [131].

Over the years, the development of the aerospace industry has led to a growing need for special light materials with high durability and resistance to fatigue. This has led to a focus on Al alloys to achieve specific specifications. Several types of Al alloys are available today [134,135,136,137], but some are more suited for aerospace applications than others. The most commonly used Al alloys in aerospace applications are outlined in Table 1 with their applications [131,138].

### 5.2. Future Perspective of Al in Aerospace Applications

Demand for Al alloys in aerospace applications is anticipated to double over the next decade. By 2025, the worldwide demand for aluminum will reach 80 million tons. As a result, the aerospace sector increasingly relies on recycled aluminum Al to meet its growing demand. In addition, there is a push for development in the materials used, the joining techniques, and the design structure of aircraft [41,141].

**Figure 21:**
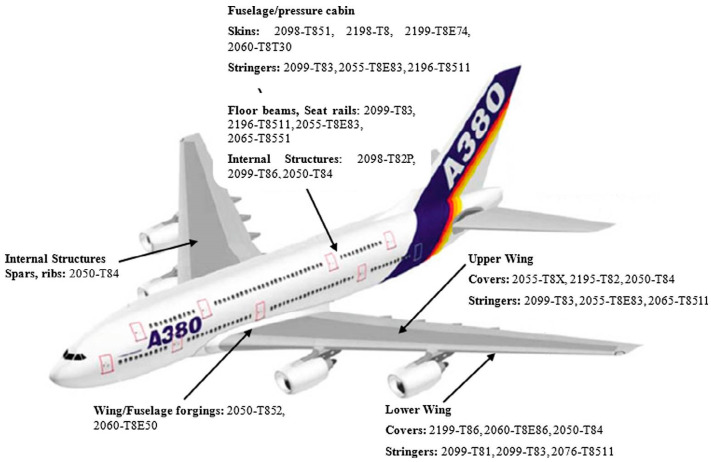
The used third generation of Al–Li alloys in the aircraft [143] (has permission from Elsevier).

Aluminum–lithium (Al–Li) alloys have been developed for use in the aerospace sector to lower aircraft weight and improve their performance. Al–Li alloys are considered new materials due to their high strength-to-weight ratio, excellent fatigue, and high toughness properties [142,143]. As more countries enter the aerospace business, there will be more development in Al–Li alloys in the years to come [41]. Table 2 lists the most popular third generation of Al–Li alloys, and Figure 21 shows the proposed use of Al–Li alloys for various aerospace applications.

Al–Cu–Li and Al–Mg–Li alloys are two of the most common types of Al–Li alloys that are used in the industry [144]. Al–Cu–Li alloys have high strength compared to the 7xxx series Al alloys and are therefore aimed to be used in high-strength engineering applications [145,146]. Al–Mg–Li alloys are extremely lightweight (density = 2.54 g/cm^3^) and exhibit a moderate strength equivalent to that of 2xxx Al alloys (except Al–Cu–Li alloys) [147,148] and Mg–Li alloys [149,150]. The AA1424 (Al–Mg–Li–Zr) Al alloy is a heat-treatable alloy that was developed out of the 1420 and 1421 alloys [144,147,148]. It gains strength from both the precipitation of intermetallic Al_3_Li and the solid-solution strengthening attainable by Mg [144,151]. Despite its welding difficulties, Al–Mg–Li alloys have garnered interest in aerospace. In addition, mechanical joinings (fasteners and riveting) are still used in the aerospace industry. FSW can be a good choice to solve most problems when combined with the fusion welding of Al–Li alloys [65,151], and to replace mechanical fasteners.

### 5.3. FSW of Conventional Al alloys

Since the invention of FSW at TWI [62,63,64], FSW has gained extensive interest from research centers, universities, and industries to identify its applicability for welding similar and dissimilar joints of different materials in various configurations. FSW possesses exceptional advantages over the traditional fusion welding method, including fewer defects, low distortion, low residual stresses, environmental friendliness, and usually excellent joint performance. Recently, it has been recognized as an ideal technology for the solid-state welding of aerospace parts made of high-strength similar [65,122,152] and dissimilar Al alloys with different thicknesses [153,154,155,156]. Additionally, weight is one of the most significant difficulties facing aircraft manufacturers. By joining Al-alloy stringers to skins for aircraft wings and fuselage components using FSW, thousands of rivets and any overlapping Al materials are eliminated. According to one renowned aircraft manufacturer, weight savings of around 1 kg/m from FSW might be realized. Aerospace producers cannot afford to ignore FSW since this welding technology can join practically any alloys—including some previously non-weldable precipitation-reinforced 2xxx and 7xxx series Al alloys [154,155,156].

During fusion welding, copper as an alloying element in the 2xxx series of Al alloys results in hot cracking, a poor solidification microstructure, and porosity in the fusion zone, which makes joining difficult. Because of this, fusion welding is not a suitable method for joining these alloy series together. Benavides et al. [157] studied the microstructural evolution during the friction stir welding of AA2024 and found that the welding process was beneficial in joining the Al 2xxx alloy type with improved mechanical properties. AA2024-T3 is a high-strength Al alloy often used in the aerospace industry. It exhibits high tensile strength, fatigue strength, a sleek surface, and low fracture spreading. It is commonly used in the exteriors of the fuselage, longitudinal beams, structures underneath wings, and sometimes in reinforcing structures and the maintenance and repair of aircraft. Sutton et al. [158] studied the variations in microstructure within a 2024-T3 Al alloy FSWed at 360 rpm and 3.3.mm/s. They concluded that the FSW could create two types of segregated, banded microstructures: hard particle-rich and particle-poor bands. The spacing of the bands was directly correlated with the welding parameters. These banded microstructures affected the macroscopic fracture process in the welds. Furthermore, by manipulating the FSW process parameters, the inhomogeneity microstructure could be avoided by increasing the fracture resistance.

The FSW approach has several potential applications in aircraft structures, especially dissimilar joints. Compared to similar welds, the dissimilar welds display a microstructure equivalent to that of similar welds but with a single lamellae flow pattern of the base materials (BM) in the SZ. This is explained by the different viscosities of the alloys during welding. Many studies are still needed to completely homogenize the mechanical properties and microstructures of the friction stir welds (FSWs) and their surrounding affected regions: the thermo-mechanical heat-affected zone (TMAZ) and the heat-affected zone (HAZ). Amancio-Filho et al. [159] investigated the effect of different rotation speeds (500–1200 rpm) and travel speeds (150–400 mm/mon) on the mechanical properties and microstructures of the dissimilar aircraft Al-alloy FSWs AA2024-T351 and AA6056-T4. The results showed that sound butt joints were obtained at the FSW parameters of 800 rpm rotation speed and 150 mm/min travel speed. This study established that in a dissimilar FSW, the weaker component determines the joint’s performance, with failure occurring in the region of strength loss due to annealing processes. Da Silva et al. [154] reported that the boundary between both BMs at the SZ for the dissimilar 2024/7075 FSWs was clearly delineated. The microstructural analysis demonstrated the formation of a recrystallized fine-grained SZ with two distinct grain sizes due to the two distinct BMs. Additionally, the threaded pin geometry also had an impact on the material flow and mixing pattern during the FSW process of the dissimilar AA2024-T3 and AA7075-T6 Al alloys. Lee et al. [153] related the strength of FSWed lap joints of AA6061 and AA5052 alloys mainly to the interface morphology and the vertical transport of each alloy material with FSW parameters. Avinash et al. [160] produced a defect-free, AA2024-T3/AA7075-T6 friction-stir-welded dissimilar butt joint at the welding parameters of 80 mm/min and 1000 rpm for the welded plate thickness ratio of 1.3. The joint strength was lower than the BMs, possibly because the dissimilar joint thickness ratio was higher than uniform. RaviKumar et al. [161] examined the dissimilar FSW of 7075-T651 and 6061-T651 Al alloys under various welding conditions: rotation speed, traversal speed, and pin geometry. They concluded that the ultimate tensile strength of 205.23 MPa was achieved at the welding parameters of 900 rpm and 10 mm/min using a taper cylindrical threaded profile. Moreover, the two material alloys were unevenly distributed in the SZ.

AA5052 provides the highest strength and ductility, making it ideal for manufacturing engine components and fittings. Additionally, it is very corrosion-resistant. AA5052-H32 is now finding applications in the aerospace industry for fabricating lightweight and low-cost TV screen frames on the back of passengers’ airplane seats. Shanavas et al. [162] studied the influences of rotational speed and travel speed on the UTS of underwater and normal FSW of 6 mm-thick AA5052-H32 aluminum alloy. It was noted that the UTS gained by underwater FSW was about 2% higher than that of the conventional FSW process. A microstructural examination revealed that the heat-affected region was not found in underwater welding [14,163,164,165]. A fractography investigation showed that all the FSWs displaying higher joint efficiency failed through ductile mode fracture.

The 6xxx series Al alloys (medium-strength aerospace alloys) are used for fuselage structures and wing skins. FSW is a viable approach for modifying the AA 6xxx alloy microstructure, resulting in a refined and homogeneous grain structure with good weld efficiency. Kumbhar and Bhanumurthy [166] investigated the effect of FSW variables on the microstructural changes and the associated mechanical performance of the AA6061-O Al alloy welded in butt joints at different rotational speeds from 710 to 1400 rpm and welding speeds from 63 to 100 mm/min. The post-weld heat treatment (PWHT) of the joints was also examined. They recommended that it is beneficial to the weld joints at lower rotational speeds and at a higher welding speed, thus improving productivity. FSW of AA6061-O increases the UTS of the welds compared to that of the BM in the O-condition for all welded joints. Moreover, PWHT for up to 8 h restores the ductility and strength while improving the microstructure homogeneities of the welds compared to that of the BM in T6. Scialpi et al. [167] studied the influence of FSW tool shoulder profiles on the microstructural and mechanical properties of FSWed AA6082-T6 Al alloy joints (1.5 mm thickness) at 1810 rpm and 460 mm/min. They found that a shoulder with a fillet and cavity worked well for thin sheets to achieve the best joints compared to the other shoulder geometries. Sato et al. [168] reported that the hardness profile across the SZ of the FSWed 6 mm-thick AA6063-T5 Al depended on the precipitate distribution and the grain size microstructure.

The 7xxx series Al alloys are one of the strongest Al alloys currently in use in the industry. A variety of aircraft structural applications benefit from its high strength-to-weight ratio and natural aging properties. Kimura et al. [169] investigated the AA 7xxx FSW joining phenomena. They concluded that the AA7075 FSW mechanism was comparable to that of low-carbon steel and attributed this phenomenon to the similarities in their strength properties. Fu et al. [170] studied the role of three ambient conditions (hot water, cold water and air) on the mechanical properties and joint efficiency of submerged 5.5 mm-thick FSW AA7050 alloys at 100 mm/min and 800 rpm in butt joints. They found that the hot-water-welded joints had the best mechanical properties of all the welded joints. Moreover, the ratio of the elongation and UTS of the joint to the BM in hot water achieved 150% and 92%, respectively. Venugopal et al. [171] investigated the microstructure and corrosion resistance of the FSW 12 mm-thick AA7075-T6 alloy welded at 350 rpm and 60 mm/min. The results showed that the pitting corrosion resistance of the welded metal (high grain refining) was better than that of TMAZ and the BM.

The available data in the published research indicate that FSW is the most suitable welding method for joining dissimilar and similar Al alloys. Friction stir welding outperforms fusion welding in strength, ductility, fatigue resistance, and fracture toughness. Ahmed et al. [172] studied the effect of varying FSW travel speeds from 50 to 200 mm/min at a constant rotation speed of 300 rpm on the mechanical properties and microstructure of similar and dissimilar AA7075-T6 and AA5083-H111 butt joints. The results showed that the applied welding parameters succeeded in producing defect-free joints. A marked grain refining was achieved in the stir zones of all the similar and dissimilar joints. The hardness profile of the similar AA7075 welds revealed typical behavior for age-hardened Al alloys with a hardness loss in the SZ, and in the case of similar AA5083 welds, typical behavior for work-hardened Al alloys with a detected enhancement in the SZ hardness. In contrast, the dissimilar welds revealed a smooth transition in the hardness profile between the two hardness values of the AA7075 and AA5083 alloys. Furthermore, the dissimilar joints showed that the UTS ranged from 245 to 267 MPa with a weld joint efficiency ranging from 77 to 87% of the strength of AA5083 BM.

### 5.4. FSW of Aluminum–Lithium Alloys

Al–Li alloys have become of great interest in the aerospace industry with the aim of reducing the structural weight of the aircraft while reducing fuel consumption. The fusion welding of Al–Li alloys results in common fusion welding defects such as hot cracks, pores, element loss and joint softening, which result in low joint strength and limit the further application of Al–Li alloys in the aerospace field [173]. Thus, FSW is one of the best methods known to eliminate these fusion welding defects as a solid-state welding process. Therefore, extensive research has been conducted to investigate the effect of FSW parameters on the microstructure and mechanical properties of different Al–Li alloys. This has been recently summarized in comprehensive reviews by Yang et al. [173] and by Mishra and Sidhar [174]. This section will summarize some of the FSW research to investigate the effect of FSW parameters on the microstructural features and mechanical properties. Wei et al. [175] investigated the effect of FSW variables in terms of pin rotational speed, travel speed, and downward force on the mechanical properties and microstructure of the FSWed AA1420 (Al–Mg–Li) alloy. They related the SZ grain size increase to the increased heat input. Furthermore, the UTS of the joints reached 86% of the BM with a 180° maximum bending angle. Sidhar et al. [151] examined the influence of post-welding treatment on the FSWs of AA1424 (Al–Mg–Li) alloy welded at 800 rpm and 305 mm/min. The results showed that the HAZ and the SZ showed a full recovery of strength. A joint efficiency of around 97% of the BM was obtained. Moreover, they ascribed the high strength of joints to high density and homogenous dispersion of the fine Al_3_Li precipitation phase. Altenkirch et al. [176] investigated the effect of FSW parameters on the residual stresses and hardness of Al–Li AA2199 during friction stir welds. They reported that the low hardness region widened with increasing downforce and tool rotation and decreased as the traversal speed increased. In terms of distortion, they reported that the conditions that reduced the heat input led to lower distortion levels.

Shukla and Baeslack [177] studied the microstructure of a friction-stir-welded thin-sheet Al–Cu–Li alloy using transmission electron microscopy. They interrelated the microhardness reduction to the dissolution and coarsening of T1 and θ′ precipitates. Additionally, Cavaliere et al. [178,179] investigated the microstructure of FSWed Al–Li 2198 alloys using TEM and obtained the same results. Steuwer et al. [180] studied the microstructure Al–Li AA2199 friction stir welds. They attributed the W-shaped hardness profile across FSW in third-generation Al–Li–Cu–Mg alloys to the dissolution of the age-hardening phases in different regions. Ma et al. [181] investigated the mechanical properties of the friction-stir-welded nugget of 2198-T8 Al–Li alloy joints. They reported that yield and tensile strength had a “U” shape through the weld zone and a lower value in the weld zone, while the elongation was reversed. De Geuser et al. [182] investigated the microstructure of a friction-stir-welded AA2050 Al–Li–Cu in the T8 state. They reported a strict correlation between the volume fraction of the T1 precipitates and the hardness of the material. Gao et al. [183] investigated the correlation between microstructure and mechanical properties in a friction-stir-welded 2198-T8 Al–Li alloy. They reported a reduction in the hardness of the weld zone and strength due to the dissolution of the T1 phase that existed in the base material. Qin et al. [184] investigated the evolution of precipitation in a friction-stir-welded 2195-T8 Al–Li alloy. Their results showed that precipitations in the base metal primarily consisted of T1 (Al_2_CuLi) platelets and small amounts of the θ′ (Al_2_Cu) and τ2 (Al_7_Cu_2_Fe) phases. In the heat-affected zone (HAZ), these precipitations dissolved during welding, allowing the re-precipitation of δ′ (Al_3_Li) and β′ (Al_3_Zr) during cooling. The δ′ and β′ phases were the primary strengthening phases in the weld nugget zone (WNZ), which resulted in the observed lower microhardness of the nugget region. Table 3 summarizes the FSW conditions and the resulting mechanical properties for Al–Li alloys. 

### 5.5. ISO Standard for Aluminum FSW

The ISO standards available for aluminum and aluminum alloys FSW are as the following:

1. ISO 25239-1:2020 Friction stir welding—Aluminum—Part 1: Vocabulary
2. ISO 25239-2:2020 Friction stir welding—Aluminum—Part 2: Design of weld joints
3. ISO 25239-3:2020 Friction stir welding—Aluminum—Part 3: Qualification of welding operators
4. ISO 25239-4:2020 Friction stir welding—Aluminum—Part 4: Specification and qualification of welding procedures
5. ISO 25239-5:2020 Friction stir welding—Aluminum—Part 5: Quality and inspection requirements

## 6. FSW of Titanium

Due to the poor thermal conductivity of titanium alloys, using conventional FSW tools results in excessive heat generation at the surface and, consequently, a significant temperature gradient across the thickness [108]. In addition, alloys such as Ti–6Al–4V have a relatively high working temperature. These two reasons have motivated the TWI to innovate a unique FSW tool known as the stationary shoulder FSW (SSFSW) tool (introduced in Section 3.2) [108,110]. The early publications in this regard were published by Wynne et al. [108], where they investigated the microstructure and texture of FSWed 6 mm-thick Ti–6Al–4V alloy. They reported that the microstructure was uniform across the thickness and significantly refined compared to the base material [108]. Zhang et al. [191] investigated the FSW of commercially pure titanium using the PCBN tool for 2 mm thickness. They reported that the SZ consisted of fine lath-shaped grains that contained PCBN debris and Ti borides. This implies the difficulty in using FSW titanium with the conventional tool even for small thicknesses. Ramulu et al. [192] evaluated the tensile properties of FSWed Ti–6Al–4V of 2 and 2.5 mm thicknesses. They reported that FSWs in the Ti–6Al–4V alloy can possess yields and ultimate tensile strengths superior to that of the parent material due to grain refinement in the weld nugget, while the decreased elongations were associated with root defects leading to premature failure in addition to the refined grain structure. Farias et al. [193] investigated WC tool wear during the FSP of Ti–6Al–4V of 2 mm thickness. They reported that the severe tool wear caused a loss of surface quality and the inclusion of fragments inside the joining, and recommended the replacement of cemented carbide with tungsten alloys. Additionally, Wang et al. [193] investigated the different FSW tool (W–1.1%La_2_O_3_ and two different grades of WC–Co-based materials) wear during the FSW of Ti–6Al–4V, and they reported that tool degradation occurred due to plastic deformation in the W–1.1%La_2_O_3_ tool. Additionally, shear-stress-induced cracks were observed at the pin tip and tool debris was left in the processed material. The mechanical fracture along with the diffusion was responsible for tool weight loss [193]. Yoon et al. [194,195,196] successfully obtained FSW joints with 5 mm-thick Ti–6Al–4V plates using a Co-based alloy tool. Due to the difficulty of obtaining FSW joints using the available tool materials, some researchers have used heat-assisting systems to obtain successful joints. For example, Ji et al. [197,198] investigated the joint formation and mechanical properties of back-heating-assisted friction-stir-welded Ti–6Al–4V. They reported that the back-heating method reduces the temperature gradient along the thickness, which is beneficial for eliminating the tearing defect; therefore, defect-free joints can be attained using a wider parameter range. Li et al. [199] designed a new FSW tool where the pin was made from W-Re25% alloy because of its excellent high-temperature wear resistance and the shoulder was made from nickel-based superalloy (GH4043) due to its good high-temperature impact property and low cost. They produced a number of defect-free welds using 2 mm-thick Ti–6Al–4V. Recently, Amirov et al. [200] obtained FSW joints with titanium (α + β) alloys using a nickel superalloy tool.

## 7. Future Challenges and Trends in FSW for Aerospace Industries

FSW as an innovative solid-state welding technology is clearly replacing the conventional welding techniques in the aerospace industry. However, the development of launch vehicle models and other aerospace applications requires the FSW community to work hard towards the development of the technology to overcome a number of limitations that hinder the extension of applying FSW to high-softening-temperature materials, composite materials, and polymeric materials. One of the key challenges that limit the use of the FSW process in high-softening-temperature materials is the tool materials that need to be cost-effective relative to conventional welding techniques. In addition, the high cost of the FSW machines needs to be continuously solved by adopting the existing CNC machines and the development of welding heads to be used within the workshop machines.

The aerospace industry needs to ensure high accuracy and high-quality manufacturing; thus, the exclusive defects of FSW joints need to be detected using advanced NDT techniques such as the phased array ultrasonic that was used during the manufacturing of the fuel tanks. Additionally, the ability to repair those defects at the highest standard and feasibility needs to be continuously developed. Provided that these shortcomings are overcome, FSW technology in conjunction with laser beam welding (LBW) will enable significant weight savings in the aerospace industry as well as in other transport systems. It is also worth pointing out that FSW technology should not be considered a competing joining process to fusion welding techniques such as LBW, but a supporting one.

## Figures and Tables

**Table 1:** The most commonly used Al alloys in aerospace applications.

Al Alloy Series	Representative Alloys	Applications
2xxx	Al clad 2024 AA2014 AA2219	Wing and fuselage sheet structures, fasteners, screws and rivets [41,134,139,140]. Aircraft internal structure, External fuel tank [41,138,140]
3xxx	AA3003, AA3005, AA3105	Air conditional tube, heat exchange Parts for aircraft engines [131,134,135]
5xxx	AA 5052	Engine components, fittings, inner body panels and structural parts [41,137,138,140]
6xxx	AA6061 AA6063	Light aircraft applications (wing and fuselage structures) Finer details of an aircraft (aesthetic and architectural finishes) [131,139,140].
7xxx	AA7050, AA7068 AA7075, AA7475	Military aircraft (wing skins and fuselage) Fuselage bulkheads of larger aircraft, aerospace applications [41,131,134,138,139,140]
8xxx	AA8009, AA8019, AA8090	Helicopter components [131,138,140]

**Table 2:** The proposed Al–Li alloys replace the traditional Al alloys in the aircraft industry [143] (has permission from Elsevier).

Al–Li Alloys	Required Property	Traditional Al Alloy	Aircraft Parts
Sheets			
2199T8E74 & 2060-T8E30 2098-T851 & 2198-T8	Medium strength Damage tolerant	2524-T351 2024-T3	Cabin skins Fuselage
Plates			
2098-T82P (sheet/plate) 2050-T84, 2055-T8X, 2195-T82 2050-T84 2195-T82/T84 2297-T87, 2397-T87 2099-T86 2199-T86, 2050-T84, 2060-T8E86	Medium strength Medium strength Medium strength High strength Medium strength High strength Damage tolerant	2024-T62 7050-T7451 2124-T851 7050-T7451, 7X75-T7XXX 7150-T7751, 7055-T7751, 7055-T7951, 7255-T7951 2024-T351, 2324-T39, 2624-T351, 2624-T39	F-16 fuselage panels Upper wing covers Spars, ribs, other internal structures Launch vehicle cryogenic tanks F-16 fuselage bulkheads Internal fuselage structures Lower wings covers
Forging			
2060-T8E50 & 2050-T852	High strength	7050-T7452 & 7175-T7351,	Wings/fuselage attachments & window and crown
Extrusions			
2099-T81, 2076-T8511 2099-T83, 2099-T81, 2196-T8511, 2055-T8E83, 2065-T8511	Damage tolerant Medium/High strength	2024-T3511, 2026-T3511, 2024-T4312 & 6110-T6511 7075-T73511, 7075-T79511, 7150-T6511,	Lower wings stringers Fuselage/Pressure cabin Fuselage/Pressure cabin Stringer and framers, upper

**Table 3:** Al–Li alloys FSW conditions and the resulting mechanical properties.

Alloy, Thickness (mm)	Tool Shape	Rotation Rate, (rpm)	Traverse Speed, (mm/min)	UTS(FSW)/UTS(BM) (%)	Hardness Profile Shape	Refs.
AA2195-T87, 5	Taper threaded pin	200–1000	100–300	390/573 (68%)–425/573 (74%)	W	[185]
AA2060-T8, 2	Cylindrical straight pin	300–1400	100	375/530 (71%)–443/530 (83%)	W	[186]
AA2198-T851 3.2	Bobbin with cylindrical pin	800	42	380/473 (80%)	W	[187]
AA2099 T8, 5	Threaded Cylindrical pin	700–1100	45	275/540 (51%)–340/540 (64%)	W	[188]
AA2099-T83, 5	threaded, tapered, triangular pin	400–1200	75–550	343/558 (61%)–390/558 (70%)	Not available	[189]
AA2050-T8, 15	Threaded pin with 3 flats	400	200	Not available	W	[182]
AA2198-T8, 2	Tapered pin	600	200	300/491 (60%)	W	[181]
AA2198-T8, 1.8	Tapered pin	800	300	386/518 (70%)	W	[183]
AA2198-T8, 3.2	Bobbin tool	400–1000	42	270/473 (57%)–380/473 (80%)	W	[190]

## Data Availability

Will be available through corresponding author.
